# Intestinal mucosal immune responses induced by novel oral poliovirus vaccine type 2 and Sabin monovalent oral poliovirus vaccine type 2: an analysis of data from four clinical trials

**DOI:** 10.1016/j.lanmic.2024.101028

**Published:** 2025-06

**Authors:** Audrey Godin, Elizabeth B Brickley, Ruth I Connor, Wendy F Wieland-Alter, Margaret E Ackerman, Joshua A Weiner, John Modlin, Minetaro Arita, Ananda S Bandyopadhyay, Chris Gast, Xavier Sáez-Llorens, Ricardo W Rüttimann, Pierre Van Damme, Ilse De Coster, Peter F Wright

**Affiliations:** a Health Equity Action Lab, Department of Infectious Disease Epidemiology and International Health, London School of Hygiene & Tropical Medicine, London, UK; b Department of Pediatrics, Geisel School of Medicine at Dartmouth, Dartmouth Health, Lebanon, NH, USA; c Thayer School of Engineering, Dartmouth College, Hanover, NH, USA; d Department of Virology II, National Institute of Infectious Diseases, Tokyo, Japan; e Bill & Melinda Gates Foundation, Seattle, WA, USA; f PATH Center for Vaccine Innovation and Access, Seattle, WA, USA; g Infectious Disease Department, Hospital del Niño Dr José Renán Esquivel, Senacyt and Cevaxin Research Centers, Panama City, Panama; h Fighting Infectious Diseases in Emerging Countries, Miami, FL, USA; i Centre for the Evaluation of Vaccination, Vaccine and Infectious Disease Institute, University of Antwerp, Antwerp, Belgium

## Abstract

**Background:**

A novel oral polio vaccine type 2 (nOPV2), which is more genetically stable (ie, lower risks of reverting to neurovirulence) than the Sabin monovalent OPV2 (mOPV2), has been deployed to interrupt circulating vaccine-derived poliovirus type 2 (PV2) outbreaks. This study compares intestinal mucosal immune responses induced by nOPV2 and mOPV2.

**Methods:**

In this analysis, we evaluated intestinal mucosal immune responses in healthy participants of different ages (ie, infants aged 18–22 weeks, children aged 1–4 years, and adults aged 18–50 years) and vaccine backgrounds (ie, OPV2-experienced *vs* OPV2-naive). Participants were selected from two phase 2 trials of nOPV2, conducted in 2018–19 (infants and children, NCT03554798 [Panama]; adults, EudraCT 2018-001684-22–NCT04544787 [Belgium]), and two phase 4 historical control trials of mOPV2, conducted in 2015–16 (infants and children, NCT02521974 [Panama]; adults, EudraCT 2015-003325-33 [Belgium]). We measured PV2-specific neutralising activity and IgA concentrations in stools collected before and 14 days after vaccination.

**Findings:**

We compared data from 160 participants (ie, 47 infants, 47 children, and 66 adults) in the nOPV2 trials to 188 participants (ie, 42 infants, 46 children, and 100 adults) in the mOPV2 trials. Within each age group, one dose of nOPV2 or mOPV2 induced similar intestinal PV2-specific neutralisation and IgA responses on day 14. Responses diminished with age: among the OPV2-naive participants who received nOPV2, 27 (82%) of 33 infants, 17 (61%) of 28 children, and four (25%) of 16 adults had detectable PV2-specific neutralisation on day 14. Despite having similar median log_10_ IgA responses (1·4 [IQR 1·0–2·2] *vs* 1·4 [1·1–1·7], p=0·34) and median log_2_ neutralisation titres (1 [IQR 1–1] *vs* 1 [1–1·5], p=0·89) on day 14, a smaller percentage of OPV2-experienced adults shed vaccine virus than OPV2-naive adults upon nOPV2 challenge (20% *vs* 82%, p<0·0001).

**Interpretation:**

We found no evidence of differences in the intestinal mucosal immune responses induced by nOPV2 or Sabin mOPV2 and observed the strongest responses in infants.

**Funding:**

The Bill & Melinda Gates Foundation, Japan Agency for Medical Research and Development.

## Introduction

Sabin oral polio vaccine (OPV), which effectively induces intestinal mucosal immunity that inhibits poliovirus types 1, 2, and 3 (PV1, PV2, and PV3) replication and limits community transmission,^1^ has been the keystone of global efforts to prevent paralytic poliomyelitis (polio).^2^ However, OPV can revert to neurovirulence, leading to cases of vaccine-associated paralytic polio and emergences of circulating vaccine-derived polioviruses (cVDPVs) in settings of low immunisation coverage.^2^ To mitigate these risks following wild PV2 eradication, routine use of type 2-containing OPV (OPV2) was stopped globally in the switch from trivalent (tOPV) to bivalent OPV (bOPV) in April, 2016.^3^ Nevertheless, cVDPV2 cases have continued to be detected,^3^^,^^4^ peaking in 2020 with 1051 cVDPV2-associated polio cases spanning 29 countries.^3^ To enhance population-level mucosal immunity and contain cVDPV2 transmission, Sabin monovalent OPV2 (mOPV2) was deployed in short-term campaigns, but its use inherently introduced risks of seeding further cVDPV2 outbreaks.^3^^,^^4^

**Research in context**

**Evidence before this study**

The international spread of polioviruses remains a WHO Public Health Emergency of International Concern. Since March, 2021, the Global Polio Eradication Initiative has deployed a novel oral polio vaccine type 2 (nOPV2) to respond to outbreaks of type 2 circulating vaccine-derived poliovirus (cVDPV2). nOPV2 was genetically engineered to have lower risk of reverting to neurovirulence than the historically used Sabin monovalent oral polio vaccine type 2 (mOPV2). Nevertheless, there has been little research investigating whether nOPV2 is as effective as mOPV2 at inducing intestinal mucosal immunity, which is crucial for inhibiting poliovirus replication and limiting transmission.

We searched PubMed without language restrictions for trials investigating the immunogenicity of nOPV2 from database inception to Aug 1, 2023, using the terms (“novel”) AND (“type 2”) AND (“OPV” OR “oral poliovirus vaccine”) AND (“immun∗”). Two publications from the four clinical trials in Panama and Belgium that used the same samples as this study reported that, as compared with mOPV2, nOPV2 was well tolerated, induced similar serum immune responses, and stimulated intestinal viral replication among infants, children, and adults. One further publication that focused on infants from the same clinical trials in Panama reported inhibited stool shedding of vaccine virus upon receipt of a second dose of either nOPV2 or mOPV2, providing preliminary evidence that one dose of nOPV2 induced primary intestinal mucosal immunity that is similar to that induced by mOPV2. Additionally, previous research from our team of a small phase 1 clinical trial in Belgium showed that nOPV2 can induce low but detectable poliovirus type 2 (PV2)-specific intestinal neutralising activity among adults. Finally, a phase 2 randomised controlled trial of nOPV2 given as a birth dose in Bangladesh found that nOPV2 was well tolerated, induced serum immunity, and did not lead to excessive shedding of vaccine virus in stool.

**Added value of this study**

To our knowledge, this is the first study to directly evaluate intestinal mucosal immunity induced by nOPV2 in comparison with Sabin mOPV2. Across three different age groups (ie, infants aged 18–22 weeks, children aged 1–4 years, and adults aged 18–50 years), we observed no evidence of a difference between nOPV2 and mOPV2 in the vaccine-induced PV2-specific neutralisation and IgA responses. Among OPV2-naive participants, we observed an inverse age-related gradient in the induction of PV2-specific neutralising activity, with lower proportions of adults than infants having detectable neutralisation 14 days after receiving nOPV2. Our findings also indicate that, as compared with their OPV2-naive peers, OPV2-experienced adults (ie, vaccinated with Sabin OPV in infancy) were less likely to shed vaccine virus in their stool when challenged with nOPV2 but developed similar magnitudes of PV2-specific neutralisation and IgA responses 2 weeks after challenge.

**Implications of all the available evidence**

This study provides evidence that nOPV2 is as effective as mOPV2 in inducing PV2-specific intestinal mucosal immunity and that vaccination induces stronger intestinal neutralising and binding antibody responses in infants than in adults. These findings suggest that the administration of nOPV2 to infants is likely to interrupt the faecal–oral transmission of PV2 and, combined with its genetic stability, to be a useful tool for interrupting cVDPV2 outbreaks, a crucial step for achieving polio eradication. Further research is needed to understand the observed age-related decline in the magnitude of intestinal mucosal immune responses and the effect of previous vaccine history in the induction of mucosal immunity across the life course. Efforts are also needed to evaluate the effectiveness of nOPV2 in populations with little pre-existing immunity and as part of cVDPV2 outbreak response.

To break this cycle, scientists genetically engineered a novel OPV2 (nOPV2) vaccine designed to have lower risks of reverting to neurovirulence.^5^ In clinical trials, nOPV2 was found to be safe and well tolerated, to induce similar serum immune responses to mOPV2,^6^, ^7^, ^8^ and to show superior genetic stability.^9^^,^^10^ Since March, 2021, approximately 1 billion doses of nOPV2 have been administered globally under a WHO Emergency Use Listing (EUL) authorisation.^5^^,^^11^ Monitoring data collected during the EUL indicates that, as compared with Sabin mOPV2, nOPV2 has had lower risks of both vaccine-associated paralytic polio^12^ and cVDPV2 emergences (ie, ten reported nOPV2-derived emergences versus an estimated 66 expected if Sabin mOPV2 was used at the same scale).^11^ On the basis of safety, effectiveness, and genetic stability data, nOPV2 received the WHO’s prequalification in December, 2023.^11^

To improve understanding of nOPV2’s potential effect on faecal–oral transmission, this study investigates whether nOPV2 and Sabin mOPV2 induce similar intestinal mucosal immunity (ie, PV2-specific neutralising activity and IgA responses) across different age groups (ie, infants aged 18–22 weeks, children aged 1–4 years, and adults aged 18–50 years) and vaccine backgrounds (ie, OPV2-experienced *vs* OPV2-naive) using stool collected during two phase 4 historical control trials of mOPV2, done before the switch in 2015–16, and two phase 2 trials of nOPV2, done in 2018–19.

## Methods

### Study design and participants

**Figure 1:**
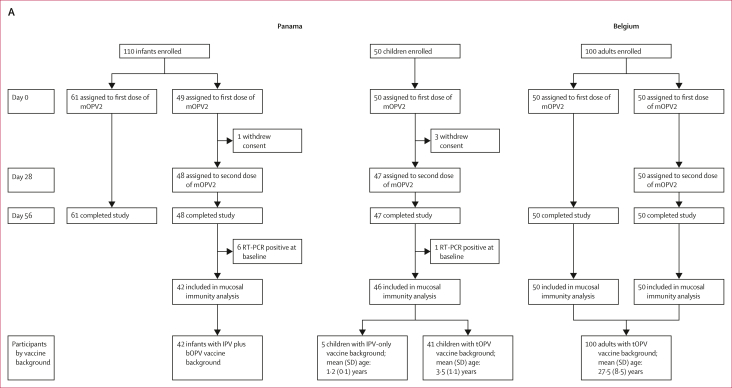

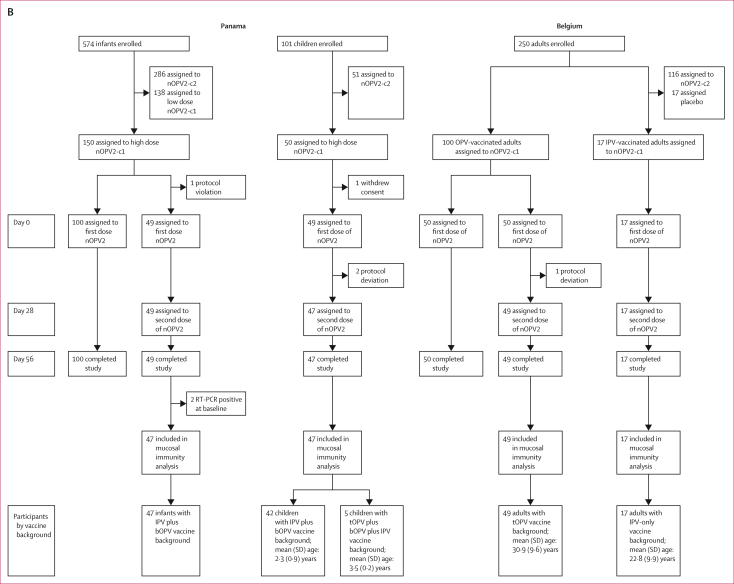
**Trial profiles of the four clinical trials included in the analytical cohort** (A) Historical mOPV2 trials (2015–16). (B) Novel OPV2 trials (2018–19). High doses contained 10^6^ 50% cell culture infectious dose; low doses contained 10^5^ 50% cell culture infectious dose. Of the two novel oral polio vaccine type 2 candidates (nOPV2-c1 and nOPV2-c2), we focused our analysis exclusively on nOPV2-c1, which is termed as nOPV2 in the accompanying text and from day 0 onwards in the flow diagram. bOPV=bivalent oral polio vaccine. IPV=inactivated polio vaccine. mOPV2=monovalent oral polio vaccine type 2. nOPV2-c1=novel oral polio vaccine type 2 candidate 1. nOPV2-c2=novel oral polio vaccine type 2 candidate 2. tOPV=trivalent oral polio vaccine.

In this analysis, we report intestinal mucosal immune responses in stools collected from participants who had received one or two doses of either nOPV2 or mOPV2 in two pairs of partially masked clinical trials^6^^,^^7^ that were designed a priori to facilitate comparisons of the safety and immunogenicity of nOPV2 and mOPV2 (figure 1). This study exclusively focuses on the induction of intestinal immunity after the first dose of nOPV2 or mOPV2 challenge to optimise comparability across all of the study groups.

One pair of clinical trials^7^ was conducted among infants and children in Panama: a phase 2 trial (NCT03554798; n=675) with nOPV2 conducted between September, 2018 and September, 2019, and a prospective historical phase 4 control trial (NCT02521974; n=160) with mOPV2 conducted between October, 2015 and April, 2016. As previously described (appendix pp 1–4), both studies included infants who received bOPV at 6, 10, and 14 weeks, inactivated polio vaccine (IPV) at 14 weeks, and were challenged with either nOPV2 or mOPV2 at 18–22 weeks of age, and children, aged 1–4 years, with a documented history of complete polio vaccination (ie, IPV-only, IPV plus bOPV, or IPV, bOPV, and tOPV in combination in the nOPV2 study, and with tOPV or IPV in the mOPV2 study). The main exclusion criteria were having received a polio vaccine within 3 months of the trial and having a family history of hereditary immunodeficiency. In the nOPV2 trial, infants received either one low dose (ie, 0·1 mL containing 10^5^ 50% cell culture infectious dose [CCID_50_]) or one high dose (ie, 1·0 mL containing 10^6^ CCID_50_), and all children received one high dose of nOPV2. To maintain comparability between the infants and children, the current analysis focused only on infants receiving the high dose of nOPV2. In the mOPV2 trial, all received one mOPV2 dose (ie, 0·1 mL containing ≥10^5^ CCID_50_).^7^

The other pair of clinical trials^6^ was conducted among adults in Belgium: a phase 2 trial (EudraCT 2018-001684-22; NCT04544787; n=250) of nOPV2 conducted between October, 2018 and February, 2019, and a prospective historical control phase 4 trial (EudraCT 2015-003325-33; n=100) with mOPV2 conducted between January and March, 2016. As previously described (appendix pp 1–4), both studies included healthy adults aged 18–50 years, with documented history of complete polio vaccination (ie, with either tOPV or IPV alone in the nOPV2 study, and with tOPV in the mOPV2 study). The main exclusion criteria were medical condition(s) likely to affect the participant’s wellbeing or immune response, pregnancy or breastfeeding, and travel to polio-endemic countries within 6 months of the trial. In the nOPV2 trial, participants were assigned to two groups on the basis of their vaccine history (ie, tOPV or IPV alone), and received one nOPV2 dose (ie, 0·3 mL containing 10^6^ CCID_50_). In the mOPV2 trial, participants received one mOPV2 dose (ie, 0·1 mL containing ≥10^5^ CCID_50_).^6^

As previously described,^6^^,^^7^^,^^13^ serum samples were collected at day 0 (ie, day of nOPV2 or mOPV2 challenge) and day 28 and sent to the United States Centers for Disease Control and Prevention (US CDC, Atlanta, GA, USA) for measurement of PV2-specific serum neutralisation. Stools were collected daily for 10 days, then weekly from days 14 to 28 and shipped frozen to the US CDC for viral shedding evaluation. In stool samples testing PV2-positive by RT-PCR, infectious poliovirus was assayed in Vero cells with titres expressed as the log_10_ CCID_50_ per gram of stool.^6^^,^^7^ Participants with PV2-positive RT-PCR results at day 0 (ie, six infants and one child in the mOPV2 study and two infants in the nOPV2 study) were excluded from all analyses.

In Panama, the trials were approved by the Hospital del Niño Dr José Renán Esquivel (Panama City, Panama) institutional review board (IRB).^7^ In Belgium, trials were approved by each study centre’s IRB (ie, Centre for the Evaluation of Vaccination, University of Antwerp–Antwerp University Hospital; and Centre for Vaccinology, Ghent University) and the Belgian regulatory authority.^6^ For all trials, written informed consent was provided by all participants or their parents or guardians. Consents included the use of the samples for further polio-related studies and the Dartmouth–Hitchcock IRB approved of sample testing and analysis for this study.

### Procedures

A subset of stool samples received by the US CDC (ie, selected to optimise the number with complete sample sets across the different age groups and vaccine backgrounds) were aliquoted and sent frozen to the Geisel School of Medicine at Dartmouth College (Hanover, NH, USA) for investigation of the intestinal mucosal immune responses. PV2-specific neutralising activity was assayed with a luciferase-expressing polio pseudovirus^14^ and defined as the reciprocal of the highest sample dilution that achieved 60% neutralisation. Undetectable neutralisation titres (ie, <4) were recorded as 2, and titres higher than the upper limit of detection (ie, >512) as 1024; results are presented as log_2_ neutralisation titres. The poliovirus type-specific IgA signal was quantified as median fluorescence intensity (MFI) by use of a multiplex assay developed by coupling monovalent IPV to fluorescently coded magnetic microspheres, as described previously.^15^, ^16^, ^17^

### Statistical analysis

Because stool samples were not available on all days for all participants, we categorised samples within windows: baseline (0–2 days), day 7 (days 5–9, ie, approximate peak of virus shedding), day 14 (days 12–16, ie, approximate peak of intestinal immune response), and day 28 (days 26–28); geometric means were calculated when multiple samples were available within a given window. Differences in the distribution of PV2-specific neutralisation and IgA responses were evaluated by use of Mann–Whitney *U* tests and Kruskal–Wallis tests. Differences in the proportions of participants with detectable virus neutralisation (ie, log_2_ stool neutralisation titres >1), and detectable viral shedding (ie, RT-PCR positive or log_10_ CCID_50_ >2·75/g, or both), for given days and ever (ie, at least one stool sample between days 1 and 28), were evaluated by use of Pearson’s χ^2^ tests. Changes in PV2-specific stool log_2_ neutralisation titre and log_10_ IgA MFI between baseline and day 14 were evaluated by use of Wilcoxon matched-pairs signed-rank tests. The induction of neutralising activity was evaluated by calculating the median fold-change in neutralisation titres (ie, the difference in log_2_ neutralisation titres between baseline and day 14) and compared by use of Mann–Whitney *U* and Kruskal–Wallis tests.

To mitigate potential confounding by vaccine background when comparing across age groups, we did subgroup analyses that restricted the data to participants with similar vaccine histories. For the nOPV2 group, we compared responses between OPV2-naive (ie, vaccinated with IPV with or without bOPV in infancy) infants, children, and adults. For the mOPV2 group, we compared responses between OPV2-experienced (ie, vaccinated with tOPV in infancy) children and adults. When differences were found by Kruskal–Wallis tests, we further investigated the differences between paired age groups (ie, infants–children, infants–adults, and children–adults) using post-hoc Dunn tests with Bonferroni adjustment.

All p values are from two-sided statistical tests. Given the multiple comparisons, we considered a p value of less than 0·01 as evidence of a statistical association. All analyses were done by use of Stata, version 17 and R, version 4.2.0.

### Role of the funding source

The Bill & Melinda Gates Foundation was involved in the study design, data interpretation and reviewing the full draft of the report but had no role in data collection, or analysis. The Japan Agency for Medical Research and Development had no role in the study design, data collection, data analysis, data interpretation, or writing of the report.

## Results

Polio type-specific intestinal mucosal immune responses were evaluated in 47 infants, 47 children, and 66 adults in the nOPV2 trials, and 42 infants, 46 children and 100 adults in the mOPV2 historical control trials (figure 1). Whereas no infants, five (11%) of 47 children, and 49 (74%) of 66 adults in the nOPV2 trials had previously received an OPV2-containing vaccine, 0 infants, 41 (89%) of 46 children and 100 (100%) of 100 adults in the mOPV2 trials had previously received tOPV vaccination (appendix p 5). Whereas all tOPV-vaccinated adults (49 [100%] of 49) in the nOPV2 group had received three previous doses of tOPV, most (88 [88%] of 100) of the tOPV-vaccinated adults in the mOPV2 group had received four previous doses of tOPV (appendix p 5).

Overall, in each age group, nOPV2 and mOPV2 induced similar PV2-specific stool neutralisation and IgA responses. Although the median PV2-specific log_2_ stool neutralisation titre was 1 (ie, undetectable) in each age group at baseline, 59 (21%) of 285 participants with stool available at baseline, including 19 (46%) of 41 children in the mOPV2 trial, had detectable PV2-specific stool neutralising activity before challenge (table 1). On day 14, PV2-specific stool neutralising activity was detected, among nOPV2 and mOPV2 recipients, in 27 (82%) of 33 and 16 (70%) of 23 infants, 17 (57%) of 30 and 15 (50%) of 30 children, and 10 (20%) of 49 and 18 (24%) of 76 tOPV-vaccinated adults, respectively (table 1).

**Table 1:** Poliovirus type 2-specific intestinal mucosal responses to the Sabin mOPV2 and nOPV2

	Infants bOPV-IPV vaccine history			Children tOPV and IPV+/-bOPV vaccine history			Adults∗ tOPV vaccine history		
	mOPV2 (n=42)	nOPV2 (n=47)	p value	mOPV2 (n=46)	nOPV2 (n=47)	p value†	mOPV2 (n=100)	nOPV2 (n=49)	p value
**Detectable neutralising activity**									
Day 0	3/33 (9%)	5/36 (14%)	0·53	19/41 (46%)	3/33 (9%)	<0·0001	17/95 (18%)	12/47 (26%)	0·29
Day 14	16/23 (70%)	27/33 (82%)	0·29	15/30 (50%)	17/30 (57%)	0·61	18/76 (24%)	10/49 (20%)	0·67
**Neutralisation log_2_ titre**									
Day 0	1 (1–1); n=33	1 (1–1); n=36	0·48	1 (1–4·0); n=41	1 (1–1); n=33	0·0008	1 (1–1); n=95	1 (1–2·1); n=47	0·30
Day 14	6·5 (1–7·9); n=23	6·4 (2·9–8·4); n=33	0·55	1·5 (1–6·1); n=30	2·8 (1–8·1); n=30	0·43	1 (1–1); n=76	1 (1–1); n=49	0·77
**Median fold change in log_2_ neutralisation titre**									
Days 0–14	3·4; n=16	4·3; n=27	0·41	1·6; n=27	2·6; n=23	0·23	0·22; n=74	−0·01; n=47	0·033
**IgA log_10_ MFI**									
Day 0	2·2 (1·7–2·6); n=27	2·4 (2·2–2·6); n=36	0·15	2·3 (1·9–3·0); n=38	1·8 (1·6–2·1); n=26	0·0016	1·3 (0·9–1·6); n=82	1·4 (1·1–2·4); n=43	0·011
Day 14	3·0 (1·9–3·5); n=19	2·9 (2·5–3·2); n=33	0·87	2·1 (1·5–3·0); n=29	2·2 (1·7–3·1); n=30	0·89	1·2 (1·0–1·7); n=68	1·4 (1·0–2·2) n=47	0·13
**Detectable log_10_ CCID_50_**‡									
Day 7	22/35 (63%)	30/43 (70%)	0·52	16/43 (37%)	38/44 (86%)	<0·0001	9/98 (9%)	10/49 (20%)	0·06
Day 14	6/24 (25%)	5/24 (21%)	0·73	4/30 (13%)	8/33 (24%)	0·27	1/78 (1%)	0/34 (0%)	0·51
Day 28	5/22 (23%)	2/12 (17%)	0·68	1/28 (4%)	6/23 (26%)	0·020	0/90 (0%)	0/25 (0%)	··
**Ever detectable log_10_ CCID_50_**‡									
Days 1–28	33/40 (83%)	34/46 (74%)	0·34	20/46 (44%)	43/47 (92%)	<0·0001	10/100 (10%)	10/49 (20%)	0·080
**Ever detectable RT-PCR**									
Days 1–28	41/42 (98%)	47/47 (100%)	0·29	42/46 (91%)	44/47 (94%)	0·67	15/100 (15%)	12/49 (25%)	0·16

Data are n/N (%) or median (IQR); p values based on Pearson’s χ^2^ or Mann–Whitney *U* tests. Because stool samples were not available on all days for all participants, we described the measurements obtained from samples collected on days 0–2 as the baseline for the stool neutralisation and IgA, on days 5–9 for day 7, on days 12–16 for day 14, and on days 26–28 for day 28. Log_2_ neutralisation titres greater than 1 were considered as detectable. Detectable log_10_ CCID_50_ does not include the lower limit of quantification (ie, 2·75). bOPV=bivalent oral polio vaccine type. CCID_50_=50% cell culture infective dose. IPV=inactivated polio vaccine. MFI=median fluorescence intensity. mOPV2=monovalent oral polio vaccine type 2. nOPV2=novel oral polio vaccine type 2. tOPV=trivalent oral polio vaccine.

∗ We excluded the adults with IPV vaccination background from the nOPV2 trial (n=17) to maintain the comparability between the groups.

† Differences between nOPV2 and mOPV2 in the children’s group might be confounded by differences in previous vaccine history (ie, higher proportions of children in the mOPV2 group had previously received tOPV vaccination compared with children in the nOPV2 group).

‡ Results might differ from previously published reports^6^^,^^7^ owing to the exclusion of stool sample data from days 29 and 30, and the inclusion of different subgroups.

**Figure 2:**
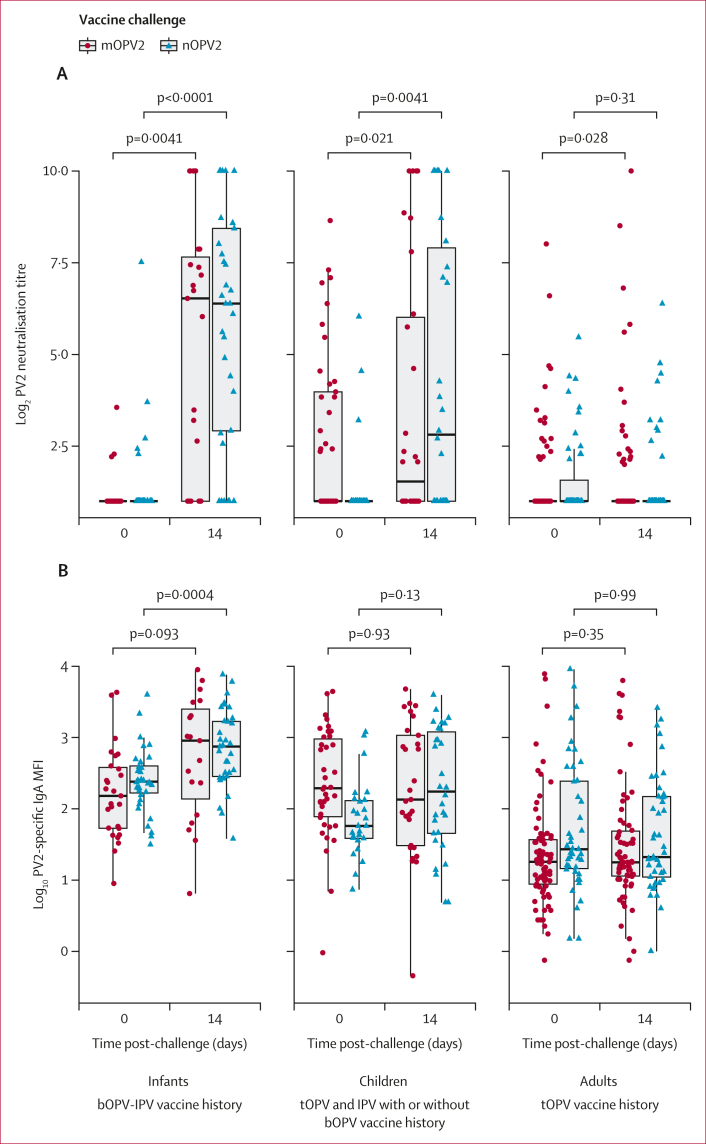
Differences in the distribution of poliovirus type 2-specific stool neutralisation titres (A) and of poliovirus type 2-specific stool IgA MFIs (B) at baseline and 2 weeks after Sabin monovalent type 2 oral poliovirus vaccine and novel type 2 oral poliovirus vaccine Colours and symbols indicate the polio vaccine received as a challenge. p values are from Wilcoxon matched-pairs signed-rank tests. Note, the adults with IPV vaccination background from the nOPV2 trial (n=17) were excluded from this analysis to maintain the comparability between the groups. bOPV=bivalent oral polio vaccine type. IPV=inactivated polio vaccine. MFI=median fluorescence intensity. mOPV2=monovalent oral polio vaccine type 2. nOPV2=novel oral polio vaccine type 2. PV2=poliovirus type 2. tOPV=trivalent oral polio vaccine type.

In infants, both the mOPV2 and nOPV2 vaccine challenge groups had similar median PV2-specific stool log_2_ neutralisation titres at baseline (mOPV2: 1 [IQR 1–1] *vs* nOPV2: 1 [1–1], p=0·48) and day 14 (6·5 [1–7·9] *vs* 6·4 [2·9–8·4], p=0·55). These groups also had similar log_10_ IgA MFIs at baseline (2·2 [1·7–2·6] *vs* 2·4 [2·2–2·6], p=0·15) and day 14 (3·0 [1·9–3·5] *vs* 2·9 [2·5–3·2], p=0·87; table 1). We observed an increase in PV2-specific stool neutralisation titres between baseline (undetectable titres) and day 14 (median titres ≥6·4) in infants receiving either nOPV2 (1 [1–1] *vs* 6·5 [1–7·9], p<0·0001) or mOPV2 (1 [1–1] *vs* 6·4 [2·9–8·4], p=0·0041; figure 2A). Although PV1-specific and PV3-specific IgA MFIs did not increase in either challenge group (appendix p 6), median PV2-specific IgA increases were observed from baseline (2·4 [2·2–2·6]) to day 14 (2·9 [2·5–3·2]) among infants in the nOPV2 group (p=0·0004; figure 2B).

In children, we observed an increase in PV2-specific stool neutralisation titres between baseline (undetectable titres) and day 14 (2·8 [1·0–8·1]) only in nOPV2 recipients (p=0·0041, figure 2A). Whereas similar PV2-specific stool neutralisation titres were attained at day 14 comparing the challenge vaccines (mOPV2: 1·5 [1–6·1] *vs* nOPV2 2·8 [1–8·1], p=0·43, table 1), the small increase in titres between baseline and day 14 in children from the mOPV2 group (baseline: 1 [1–4·0] *vs* day 14: 1·5 [1–6·1], p=0·021, figure 2A) might be explained by higher titres than the nOPV2 group at baseline in this group (p=0·0008; table 1) and because most of these children (41 [89%] of 46) had previously received tOPV. Similarly, although higher PV2-specific log_10_ IgA MFIs were observed at baseline in the mOPV2 group compared to the nOPV2 group (mOPV2: 2·3 [1·9–3·0] *vs* nOPV2: 1·8 [1·6–2·1], p=0·0016), PV2-specific IgA responses at day 14 were similar between these groups (2·1 [1·5–3·0] *vs* 2·2 [1·7–3·1], p=0·89, table 1).

Among tOPV-vaccinated adults, we observed no increase in PV2-specific stool neutralisation titres or IgA MFIs between baseline and day 14 in the mOPV2 group (neutralisation day 0: 1 [1–1] *vs* day 14: 1 [1–1], p=0·028; IgA day 0: 1·3 [0·9–1·6] *vs* day 14: 1·2 [1·0–1·7], p=0·35) or the nOPV2 group (neutralisation day 0: 1 [1–2·1] *vs* day 14: 1 [1–1], p=0·31; IgA day 0: 1·4 [1·1–2·4] *vs* day 14: 1·4 [1·0–2·2], p=0·99); table 1, figure 2A).

Of note, PV2-specific stool neutralisation titres and IgA MFI measured on day 14 were strongly positively correlated in the nOPV2 group in infants and children (Spearman’s ρ=0·75 in infants, 0·88 in children, p<0·0001 for both) and in the adults from both challenge groups (in the nOPV2 group, Spearman’s ρ=0·49, p=0·0005; in the mOPV2 group, ρ=0·65, p<0·0001; appendix pp 7–8).

Among OPV2-naive participants challenged with nOPV2, we observed an age-related decline in the proportion with detectable PV2-specific neutralisation and the magnitudes of neutralising activity and IgA MFIs. Within this group, 27 (82%) of 33 infants, 17 (61%) of 28 children, and four (25%) of 16 adults had detectable PV2-specific neutralisation on day 14 (table 2). Further, the median (IQR) PV2-specific log_2_ neutralisation titres at day 14 were 6·4 (2·9–8·4) in infants, 3·2 (1–8·4) in children, and 1 (1–1·5) in adults. The median difference of the log_2_ PV2-specific neutralisation titres between baseline and day 14 (fold change) were 4·3 in infants, 2·9 in children, and 0·3 in adults (table 2). Pairwise comparisons showed significant differences in the fold changes of PV2-specific log_2_ neutralisation between adults and infants (p=0·0002), but not between adults and children (p=0·02), nor between infants and children (p=0·26). The median (IQR) IgA MFIs were higher in the infant group than in the adults at baseline (infants: 2·4 [2·2–2·6] *vs* adults: 1·1 [1·0–1·4], p<0·0001) and day 14 (infants: 2·9 [2·5–3·2] *vs* adults: 1·4 [1·1–1·7], p<0·0001; table 2).

**Table 2:** Poliovirus type 2-specific intestinal mucosal responses to the novel type 2 oral poliovirus vaccine in OPV2-naive (ie, IPV with or without bOPV-vaccination history) participants

	Infants, n=47	Children, n=42	Adults, n=17	p value
**Detectable neutralising activity**				
Day 0	5/36 (14%)	2/29 (7%)	2/17 (12%)	0·67
Day 14	27/33 (82%)	17/28 (61%)	4/16 (25%)	0·001
**Neutralisation log_2_ titre**				
Day 0	1 (1–1); n=36	1 (1–1); n=29	1 (1–1); n=17	0·68
14	6·4 (2·9–8·4); n=33	3·2 (1-8·4); n=28	1 (1–1·5); n=16	0·0002∗
**Fold change in log_2_ neutralisation titre**				
Days 0–14	4·3; n=27	2·9; n=21	0·3; n=16	0·0008†
**IgA log_10_ MFI**				
Day 0	2·4 (2·2–2·6); n=36	1·8 (1·6–2·1); n=23	1·1 (1·0–1·4); n=14	0·0001‡
Day 14	2·9 (2·5–3·2); n=33	2·3 (1·6–3·1); n=28	1·4 (1·1–1·7); n=15	0·0001§
**Ever detectable log_10_ CCID_50_**				
Days 1–28	34/46 (74%)	41/42 (98%)	14/17 (82%)	0·008
**Ever detectable RT-PCR**				
Days 1–28	47/47 (100%)	41/42 (98%)	14/17 (82%)	0·004

Data are n/N (%) or median (IQR). p values based on Pearson’s χ^2^, Kruskal–Wallis, or Mann–Whitney *U* tests. Because stool samples were not available on all days for all participants, we described the measurements obtained from samples collected on days 0–2 as the baseline for the stool neutralisation and IgA, on days 12–16 for day 14, and on days 26–28 for day 28. Log_2_ neutralisation titres greater than 1 were considered as detectable. Detectable log_10_ CCID_50_ does not include the lower limit of quantification (ie, 2·75). bOPV=bivalent oral polio vaccine. CCID_50_=50% cell culture infective dose. IPV=inactivated polio vaccine. MFI=median fluorescence intensity. OPV2=oral polio vaccine type 2.

∗ Dunn tests 2×2 age categories p<0·0001 between adults and infants, p=0·005 between adults and children and p=0·24 between infants and children.

† Dunn tests 2×2 age categories p=0·0002 between adults and infants, p=0·02 between adults and children, and p=0·26 between infants and children.

‡ Dunn tests 2×2 age categories p<0·0001 between adults and infants, p=0·009 between adults and children and p=0·0002 between infants and children.

§ Dunn tests 2×2 age categories p<0·0001 between adults and infants, p=0·006 between adults and children and p=0·03 between infants and children.

Among OPV2-experienced participants challenged with mOPV2, the median IgA MFIs were higher in children than adults at baseline (children: 2·3 [1·9–3·0] *vs* adults: 1·3 [0·9–1·6], p<0·0001) and day 14 (children: 2·1 [1·5–2·9] *vs* adults: 1·2 [1·0–1·7], p<0·0001; table 3), which might reflect differences in the duration of time since tOPV receipt or age-related differences.

**Table 3:** Poliovirus type 2-specific intestinal mucosal responses to the Sabin mOPV2 in OPV2-experienced (ie, tOPV-vaccinated) participants

	Infants, n=0	Children, n=41	Adults, n=100	p value
**Detectable neutralising activity**				
Day 0	··	15/36 (42%)	17/95 (18%)	0·005
Day 14	··	12/27 (44%)	18/76 (24%)	0·041
**Neutralisation log_2_ titre**				
Day 0	··	1 (1 –3·6); n=36	1 (1–1); n=95	0·0023
Day 14	··	1 (1–4·6); n=27	1 (1–1); n=76	0·026
**Fold change in log_2_ neutralisation titre**				
Days 0–14	··	1·1; n=24	0·2; n=74	0·23
**IgA log_10_MFI**				
Day 0	··	2·3 (1·9–3·0); n=33	1·3 (0·9–1·6); n=82	<0·0001
Day 14	··	2·1 (1·5–2·9); n=27	1·2 (1·0–1·7); n=68	<0·0001
**Ever detectable log_10_ CCID_50_**				
Days 1–28	··	15/41 (37%)	10/100 (10%)	<0·0001
**Ever detectable RT-PCR**				
Days 1–28	··	37/41 (90%)	15/100 (15%)	<0·0001

Data are n/N (%) or median (IQR). p values based on Pearson’s χ^2^, or Mann–Whitney *U* tests. Because stool samples were not available on all days for all participants, we described the measurements obtained from samples collected on days 0–2 as the baseline for the stool neutralisation and IgA, on days 12–16 for day 14, and on days 26–28 for day 28. Log_2_ neutralisation titers greater than 1 were considered as detectable. Detectable log_10_ CCID_50_ does not include the lower limit of quantification (ie, 2·75). CCID_50_=50% cell culture infective dose. MFI=median fluorescence intensity. mOPV2=monovalent oral polio vaccine type 2. OPV2=oral polio vaccine type 2. tOPV=trivalent oral polio vaccine.

We observed no differences between nOPV2 and mOPV2 in the patterns of vaccine viral shedding (ie, both detection by RT-PCR and replication in culture) in stool collected after challenge in infants and tOPV-vaccinated adults (table 1). In the children’s age group, we observed a greater percentage of detectable viral shedding (ie, log_10_ CCID_50_ titres >2·75/g) in the nOPV2 recipients (43 [92%] of 47) compared with the mOPV2 recipients (20 [44%] of 46; p<0·0001). This difference might be explained by their vaccine background since most children (41 [89%] of 46) in the mOPV2 group had received at least three tOPV doses whereas only five (11%) of 47 children in the nOPV2 group had received a single dose of tOPV.

When analysing viral shedding by vaccine history in adults who received nOPV2 (table 4), we observed a lower proportion of individuals with detectable virus replication among OPV2-experienced participants (10 [20%] of 49) as compared with the OPV2-naive participants (14 [82%] of 17; p<0·0001). However, PV2-specific neutralising activity and IgA responses measured in stools at day 14 did not differ by vaccine background (p>0·34) and were consistently low in both groups (only 25% of IPV-vaccinated adults and 20% of tOPV-vaccinated adults had detectable PV2-specific neutralisation on day 14).

**Table 4:** Poliovirus type 2-specific intestinal mucosal responses to the nOPV2 in adults, comparing OPV2-naive (ie, IPV-vaccinated) versus OPV2-experienced (ie, tOPV-vaccinated) participants

	IPV-only vaccine history (n=17)	tOPV vaccine history (n=49)	p value
**Detectable neutralising activity**			
Day 0	2/17 (12%)	12/47 (26%)	0·24
Day 14	4/16 (25%)	10/49 (20%)	0·70
**Neutralisation log_2_ titre**			
Day 0	1 (1–1); n=17	1 (1–2·1); n=47	0·16
Day 14	1 (1–1·5); n=16	1 (1–1); n=49	0·89
**IgA log_10_MFI**			
Day 0	1·1 (1·0–1·4); n=14	1·4 (1·1–2·4); n=47	0·020
Day 14	1·4 (1·1–1·7); n=15	1·4 (1·0–2·2); n=47	0·34
**Detectable log_10_ CCID_50_**			
Day 7	14/17 (82%)	10/49 (20%)	<0·0001
Day 14	2/9 (22%)	0/34 (0%)	0·005
Day 28	0/7 (0%)	0/25 (0%)	··
**Ever detectable log_10_ CCID_50_**			
Days 1–28	14/17 (82%)	10/49 (20%)	<0·0001
**Ever detectable RT-PCR**			
Days 1–28	14/17 (82%)	12/49 (24%)	<0·0001

p values based on Pearson’s χ^2^ or Mann–Whitney *U* tests. Because stool samples were not available on all days for all participants, we described the measurements obtained from samples collected on days 0–2 as the baseline for stool neutralisation and IgA, on days 5–9 for day 7, on days 12–16 for day 14, and on days 26–28 for day 28. Log_2_ neutralisation titres greater than 1 were considered as detectable. Detectable log_10_ CCID_50_ does not include the lower limit of quantification (ie, 2·75). CCID_50_=50% cell culture infective dose. IPV=inactivated polio vaccine. MFI=median fluorescence intensity. nOPV2=novel oral polio vaccine type 2. OPV2=oral polio vaccine 2. tOPV=trivalent oral polio vaccine.

## Discussion

We assessed the intestinal mucosal immune responses after one dose of nOPV2 or Sabin mOPV2 in stool samples from healthy fully vaccinated infants, children, and adults. Within each age group, the PV2-specific stool neutralising activity and IgA responses induced 14 days post-challenge did not differ between nOPV2 and mOPV2 recipients. Among OPV2-naive participants, we observed an inverse age-related gradient in the induction of PV2-specific intestinal mucosal immune responses, with more robust responses in infants than in adults. Further, as compared with their OPV2-naive peers, OPV2-experienced adults (ie, vaccinated with tOPV in infancy) were less likely to shed vaccine virus when challenged with nOPV2 but developed similar magnitudes of PV2-specific neutralisation and IgA responses on day 14.

Knowledge regarding the induction of intestinal mucosal immunity by nOPV2 is still growing. Our results align with a phase 1 trial of nOPV2, which showed a modest but detectable intestinal PV2-specific neutralising response to nOPV2 among healthy adults^18^ and supplements the previously reported evidence of similar shedding patterns after a first dose of nOPV2 or mOPV2 in infants with reductions in shedding after second doses of each vaccine, consistent with the induction of primary intestinal mucosal immunity.^19^ No evidence of a difference in the effectiveness of nOPV2 and mOPV2 has been observed in a retrospective case–control study from 2017 to 2022 in Nigeria.^20^ Further, high seroconversion rates have been reported after nOPV2 campaigns following cVDPV2 outbreaks in 2021 in Tajikistan, and in 2021–22 in The Gambia.^21^^,^^22^ By contrast, a serological survey done in Liberia in 2021 reported unexpectedly low PV2 seroprevalence after two nOPV2 campaigns,^23^ potentially owing to recall bias from parental reporting of doses and the high prevalence of intestinal infections. Additionally, in a randomised controlled trial in Bangladesh, a lower PV2-specific serum immunogenicity was observed after co-administration of nOPV2 and bOPV in polio vaccine-naive infants in comparison with the administration of nOPV2 alone.^24^ Although our results showing strong and largely equivalent induction of intestinal immunity between nOPV2 and mOPV2 in the target infant age group are encouraging, further work is needed to evaluate the intestinal mucosal immune responses induced by nOPV2 in the context of outbreaks, low-income settings, or co-administration with other oral polio vaccines.

We have previously reported, using the same immunoassays, robust induction of PV2-specific neutralising activity and IgA in stools collected 2 weeks after a single challenge dose of mOPV2 in infants previously immunised with IPV plus bOPV,^15^ IPV only^15^, or bOPV plus monovalent high-dose type 2–specific IPV.^25^ However, we observed only low intestinal mucosal immune response after mOPV2 challenge in IPV-vaccinated children aged 1–5 years in a 2016 trial in Lithuania,^26^ and very modest homologous PV serotype-specific intestinal neutralisation in IPV-vaccinated adults after mOPV1 challenge in a 2011 trial in Sweden^17^ and after nOPV2 challenge in a 2017 trial in Belgium.^18^ Our observation of an age-related gradient in the magnitude of intestinal mucosal responses to nOPV2 reinforces the hypothesis of a diminution of the intestinal IgA response to poliovirus that develops through childhood, possibly by an age-related decrease of IgA production or degradation of IgA while transiting through the adult intestinal tract.^1^ It raises the question of whether the intestinal mucosal response to poliovirus is restricted in older children and adults or whether it might not be IgA-mediated.

The observed reduced viral shedding among tOPV-vaccinated adults compared with IPV-vaccinated adults aligns with current knowledge that previous IPV vaccination has little effect on reducing stool shedding after OPV challenge,^25^^,^^27^, ^28^, ^29^, ^30^ whereas previous exposure to OPV in infancy induces enteric immunity and limits viral shedding after homotypic oral challenge.^1^^,^^27^^,^^28^ As tOPV-vaccinated adults were able to inhibit shedding upon challenge despite having low PV2-specific neutralisation and IgA MFIs at baseline, this suggests that alternative immune mechanisms in the mucosa might contribute to controlling the intestinal poliovirus replication and shedding in adulthood.^1^ Furthermore, among OPV2-naive participants, only infants and children had substantial induction of mucosal responses after nOPV2 challenge. These results suggest that adults who only received IPV in infancy, while being seroprotected, have the potential to contribute to silent poliovirus transmission, and that an additional dose of live vaccine might be less efficient in adults than infants in stimulating intestinal mucosal immunity.

Although this study provides valuable information on vaccine-induced mucosal immunity to poliovirus in different age groups and vaccine histories, there are limitations. One main limitation was the time gap between the mOPV2 trials done in 2015–16 and the nOPV2 trials in 2018–19. Although the trials were specifically designed to compare both vaccines by use of the same protocol and analysis, residual confounding factors might bias the observed associations. Further, in the mOPV2 trials, done before the cessation of using OPV2-containing vaccines, participants were more likely to be environmentally exposed to OPV2. Second, the multiple comparisons were a concern; however, we remained cautious in our conclusions, and the consistency of the findings across subgroups suggests that they are unlikely to be a chance finding. Third, the lack of available stool after day 14 has prevented the study of the duration of mucosal immunity. Finally, the study was done in fully vaccinated healthy infants, young children, and adults. The results might not be generalisable to immunodeficient individuals, older children or adolescents, or unvaccinated or incompletely immunised individuals. Similarly, the results might not be generalisable to countries with differing socioeconomic conditions.

This study's findings affirm the utility of nOPV2 as an important tool to address the ongoing cVDPV2 outbreaks. Further research is needed to understand the effect of age and previous immunisation on poliovirus vaccine-induced mucosal immunity and to inform policy makers of the optimal age target for reducing poliovirus transmission in supply-limited outbreak settings. The evaluation of mucosal immunity after nOPV2 vaccination in low-income countries having cVDPV2s outbreaks and among individuals who are not fully vaccinated is crucial for understanding the effectiveness of nOPV2 in cVDPV2 outbreaks.

## Data sharing

De-identified participant data for this study will be made available to other investigators on reasonable request.

## Declaration of interests

All authors declare no competing interests.
