# Supplementary material for: Intestinal mucosal immune responses induced by novel oral poliovirus vaccine type 2 and Sabin monovalent oral poliovirus vaccine type 2: an analysis of data from four clinical trials
Source: Lancet Microbe. 2025 Jun;6(6):None. doi: 10.1016/j.lanmic.2024.101028 (PMC12134050; doi:10.1016/j.lanmic.2024.101028)

THE LANCET
Microbe

Supplementary appendix

This appendix formed part of the original submission and has been peer reviewed.
We post it as supplied by the authors.

Supplement to: Godin A, Brickley EB, Connor RI, et al. Intestinal mucosal immune responses induced by novel oral poliovirus vaccine type 2 and Sabin monovalent oral poliovirus vaccine type 2: an analysis of data from four clinical trials. *Lancet Microbe* 2025. https://doi.org/10.1016/j.lanmic.2024.101028

Supplementary appendix

Table 1. Inclusion and exclusion criteria from the four clinical trials. 1

Table 2. Demographics of the study population........ 5

Figure 1. Differences in the distribution of poliovirus type 1 and 3-specific stool IgA MFIs at baseline and two weeks after monovalent polio vaccine type 2 challenge........ 6

Figure 2. Correlations between serum responses, viral shedding, and markers of mucosal immunity. 7

Table 1. Inclusion and exclusion criteria from the four clinical trials.

	Inclusion Criteria	Exclusion criteria
**Adults (Belgium) mOPV2 trial**	1. Healthy male or female, between 18 and 50 years old, extremes included;<br>2. Received at least 3 doses of OPV in the past (more than 12 months before the start of the study);<br>3. In good physical and mental health as determined on the basis of medical history and general physical examination performed at Day 0;<br>4. Female subjects of childbearing potential must agree to the use of an effective method of birth control throughout the study and up to 3 months after last vaccine dose;<br>5. Willing to adhere to the prohibitions and restrictions specified in this protocol;<br>6. Informed Consent Form (ICF) signed voluntarily by the subject before any study-related procedure is performed, indicating that the subject understands the purpose of and procedures required for the study and is willing to participate in the study.	1. A condition that, in the opinion of the Investigator, could compromise the well-being of the subject or course of the study, or prevent the subject from meeting or performing any study requirements;<br>2. Having Crohn's disease or ulcerative colitis or having had major surgery of the gastrointestinal tract involving significant loss or resection of the bowel;<br>3. A known allergy, hypersensitivity, or intolerance to the study vaccine, or to any of its components, or to any antibiotics;<br>4. Any confirmed or suspected immunosuppressive or immunodeficiency condition (including human immunodeficiency virus [HIV] infection);<br>5. Will have household or professional contact with known immunosuppressed people or people without full polio vaccination (i.e. complete priming) within 28 days after vaccination;<br>6. Neonatal nurses or others having professional contact with children under 6 months old within 28 days after vaccination;<br>7. Chronic administration (i.e., longer than 14 days) of immunosuppressant drugs or other immune-modifying drugs within 6 months prior to the first vaccine dose or planned use during the study. For instance, for corticosteroids, this means prednisone, or equivalent, ≥ 0.5 mg/kg/day (inhaled and topical steroids are allowed, whereas intra-articular and epidural injection/administration of steroids are not allowed);<br>8. Presence of contraindications to administration of the study vaccine on Day 0: acute severe febrile illness deemed by the Investigator to be a contraindication for vaccination or persistent diarrhea or vomiting;<br>9. Indications of drug abuse or excessive use of alcohol at Day 0;<br>10. Being pregnant or breastfeeding. Women of childbearing potential will undergo a urine pregnancy test at Day 0. Subjects with a positive pregnancy test will be excluded;<br>11. Participation in another clinical study within 28 days prior to entry in this study or receipt of any investigational product (drug or vaccine) other than the study vaccine within 28 days prior to the first administration of study vaccine, or planned use during the study period;<br>12. Planned administration of any vaccine other than the study vaccine within 28 days of the first dose of study vaccine and during the entire study period.<br>13. Administration of polio vaccine within 12 months before the start of the study.<br>14. Having had a transfusion of any blood product or application of immunoglobulins within the 4 weeks prior to the first administration of study drugs or during the study.<br>15. Subject is an employee of the Investigator or study site, with direct involvement in the proposed study or

		other studies under the direction of that Investigator or study site, or is a family member of an employee or the Investigator.
**Adults (Belgium) nOPV2 trial**	1. For tOPV vaccinated group: healthy males or females, from 18 to 50 years of age inclusive, having previously received at least 3 doses of OPV more than 12 months before the start of the study;<br>2. For IPV-only vaccinated group: healthy males or females, from 18 to 50 years of age inclusive, having previously received at least 3 doses of IPV more than 12 months before the start of the study;<br>3. Having residence in Belgium;<br>4. In good physical and mental health as determined on the basis of medical history and general physical examination performed at Day 0;<br>5. Female subjects of childbearing potential must agree to the use of an effective method of birth control throughout the study and up to 3 months after last vaccine dose;<br>6. Willing to adhere to the prohibitions and restrictions specified in this protocol;<br>7. Informed Consent Form (ICF) and Code of Conduct signed voluntarily by the subject before any study-related procedure is performed, indicating that the subject understands the purpose of any procedures required for the study and is willing to participate in the study.	1. A condition that, in the opinion of the Investigator, could compromise the well-being of the subject or course of the study, or prevent the subject from meeting or performing any study requirements;<br>2. For IPV-only vaccinated group: ever having received any OPV in the past;<br>3. Any travel to polio endemic countries or countries with evidence of recent (within last 6 months) wild or vaccine-derived poliovirus circulation during the total duration of the study;<br>4. Professional handling of food, catering or food production activities during the total duration of the study;<br>5. Having Crohn's disease or ulcerative colitis or having had major surgery of the gastrointestinal tract involving significant loss or resection of the bowel;<br>6. A known allergy, hypersensitivity, or intolerance to the study vaccine or the placebo, or to any of their components or to any antibiotics;<br>7. Any confirmed or suspected immunosuppressive or immunodeficiency condition (including human immunodeficiency virus [HIV] infection, hepatitis B or C infections or total serum IgA level below lab lower limit of normal);<br>8. Will have household or professional contact with known immunosuppressed people or people without full polio vaccination (i.e. complete primary infant immunization series), e.g. babysitting during the total duration of the study;<br>9. Neonatal nurses or others having professional contact with children under 6 months old during the total duration of the study;<br>10. Chronic administration (i.e., longer than 14 days) of immunosuppressant drugs or other immune-modifying drugs within 6 months prior to the first vaccine dose or planned use during the study. For instance, for corticosteroids, this means prednisone, or equivalent, ≥ 0.5 mg/kg/day (inhaled and topical steroids are allowed whereas intra-articular and epidural injection/administration of steroids are not allowed);<br>11. Presence of contraindications to administration of the study vaccine on Day 0: acute severe febrile illness deemed by the Investigator to be a contraindication for vaccination or persistent diarrhea or vomiting;<br>12. Indications of drug abuse or excessive use of alcohol at Day 0 (males: > 21 units per week (m); females > 14 units per week);<br>13. Being pregnant or breastfeeding. Women of childbearing potential will undergo a urine pregnancy test at each vaccination visit. Subjects with a positive pregnancy test will be excluded;<br>14. Participation in another clinical study within 28 days prior to entry in this study or receipt of any

		investigational product (drug or vaccine) other than the study vaccine within 28 days prior to the first administration of study vaccine, or planned use during the study period;<br>15. Administration of any vaccine other than the study vaccine within 28 days prior to the first dose of study vaccine and during the entire study period;<br>16. Administration of any polio vaccine within 12 months before the start of the study;<br>17. Having had a transfusion of any blood product or application of immunoglobulins within the 4 weeks prior to the first administration of study vaccine or during the study;<br>18. Subject is an employee of the Investigator or study site, with direct involvement in the proposed study or other studies under the direction of that Investigator or study site, or is a family member of an employee or the Investigator, or was a study subject in the historical control studies UAM1 or UAT1 or in the study UAM4a;<br>19. Having a family or household member participating in the study CVIA 065 or being a study subject in the study CVIA 065.
**Infants and children (Panama) mOPV2 trial**	1. Children aged 1 to 5 years previously vaccinated with three or four doses of IPV or unvaccinated infants aged 6 weeks (-7 to +14 days).<br>2. For infants (enrolled at 6 weeks of age): must have been vaccinated with 3 doses of bOPV and one dose of IPV prior to administration of the study vaccine, and the last IPV dose must have been administered at least 4 weeks prior to the study vaccine.<br>3. Healthy without obvious medical conditions that preclude the subject to be in the study as established by the medical history and physical examination.<br>4. Written informed consent obtained from 1 or 2 parent(s) or legal guardian(s) as per country regulations.	1. For Infants: polio vaccines within the 3 months prior to the administration of the study vaccine (number of previous polio vaccine doses to be documented). For children: polio vaccines prior to administration of the study vaccine other than 3 doses of bOPV and 1 dose of IPV.<br>2. Any confirmed or suspected immunosuppressive or known immunodeficient condition including human immunodeficiency virus (HIV) infection.<br>3. Family history of congenital or hereditary immunodeficiency.<br>4. Major congenital defects or serious uncontrolled chronic illness (neurologic, pulmonary, gastrointestinal, hepatic, renal, or endocrine).<br>5. Known allergy to any component of the study vaccines or to any antibiotics.<br>6. Uncontrolled coagulopathy or blood disorder contraindicating intramuscular injections (of IPV).<br>7. Administration of immunoglobulins and/or any blood products since birth or planned administration during the study period.<br>8. Acute severe febrile illness at day of vaccination deemed by the Investigator to be a contraindication for vaccination (the child can be included at a later time if within age window and all in/exclusion criteria are met.).<br>9. Member of the subject's household (living in the same house or apartment unit) has received OPV in the last 3 months.<br>10. Subject who, in the opinion of the Investigator, is unlikely to comply with the protocol or is inappropriate to be included in the study for the safety or the benefit-risk ratio of the subject.

**Infants and children (Panama) nOPV2 trial**	1. Children enrolled at 1 to 5 years of age who have previously been fully vaccinated according to MoH recommendations with OPV and/or IPV.<br>2. Infants enrolled at 6 weeks of age (-1, + 2 weeks) with birth weight >2,500 gm. To be eligible to continue into the experimental phase of the study infants must be vaccinated with 3 doses of bOPV and one dose of IPV prior to administration of the study vaccine at 18–22 weeks of age to take into account the visit windows for enrollment (age 6 weeks, -1 or + 2 weeks) and subsequent OPV vaccination windows (± 1 week). The last polio vaccine must have been administered at least 4 weeks prior to the first dose of study vaccine.<br>3. Healthy children without obvious medical conditions like immunodeficiency diseases, severe congenital malformations, severe neurological diseases or any other disease that require high doses of corticosteroids or immunotherapies that preclude the subject to be in the study as established by the medical history and physical examination.<br>4. Written informed consent obtained from 1 or 2 parent(s) or legal guardian(s) as per country regulations.	1. For all participants the presence of anyone under 10 years of age in the subject's household (living in the same house or apartment unit) who does not have complete "age appropriate" vaccination status with respect to poliovirus vaccines at the time of study vaccine administration. For household members younger than 18 months age appropriate vaccination is at least three (3) doses of IPV. For household members between 18 months and 10 years "age appropriate" vaccination is at least three (3) doses of IPV or tOPV plus one (1) booster dose of any antipolio vaccine.<br>2. For all participants having a member of the subject's household (living in the same house or apartment unit) who is under 6 months of age at the moment of study vaccine administration.<br>3. For all participants having a member of the subject's household (living in the same house or apartment unit) who has received OPV in the previous 3 months before study vaccine administration.<br>4. For children: receipt of polio vaccines within the 3 months prior to the administration of the study vaccine (number of previous polio vaccine doses to be documented). Any other vaccine 4 weeks before study entry.<br>5. For children: any participating children attending day care or pre-school during their participation in the study until one month after their last nOPV2 administration.<br>6. For infants: any receipt of polio vaccines prior to administration of the study vaccine other than 3 doses of bOPV and 1 dose of IPV.<br>7. Any confirmed or suspected immunosuppressive or known immunodeficient condition including human immunodeficiency virus (HIV) infection in the potential participant or any member of the subject's household.<br>8. Family history of congenital or hereditary immunodeficiency.<br>9. Major congenital defects or serious uncontrolled chronic illness (neurologic, pulmonary, gastrointestinal, hepatic, renal, or endocrine).<br>10. Known allergy to any component of the study vaccines or to any antibiotics, that share molecular composition with the nOPV2 vaccines.<br>11. Uncontrolled coagulopathy or blood disorder contraindicating intramuscular injections (of IPV).<br>12. Administration of immunoglobulins and/or any blood products since birth or planned administration during the study period.<br>13. Acute severe febrile illness at day of vaccination deemed by the Investigator to be a contraindication for vaccination (the child can be included at a later time if within age window and all inclusion criteria are met.).<br>14. Subject who, in the opinion of the Investigator, is unlikely to comply with the protocol or is inappropriate to be included in the study for the safety or the benefit-risk ratio of the subject.

Table 2. Demographics of the study population.

	INFANTS n (%)		CHILDREN Mean (Sd), n (%)		ADULTS Mean (Sd), n (%)		
	mOPV2 (n=42)	nOPV2 (n=47)	mOPV2 (n=46)	nOPV2 (n=47)	mOPV2 (n=100)	nOPV2 IPV background (n=17)	nOPV2 tOPV background (n=49)
**Age (year)**	--	--	3.2 (1.2)	2.5 (1.0)	27.5 (8.5)	22.8 (9.9)	30.9 (9.58)
**Sex female**	16 (38.1%)	28 (59.6%)	28 (60.9%)	23 (48.9%)	56 (56.0%)	11 (64.7%)	29 (59.2%)
**Previous polio vaccination history**							
3 IPV or more only	0	0	5 (10.9%)	13 (27.7%)	0	17 (100%)	0
IPV + bOPV	42 (100%)	47 (100%)	0	29 (61.7%)	0	0	0
4 tOPV	0	0	34 (73.9%)	0	88 (88.0%)	0	0
3 tOPV	0	0	7 (15.2%)	0	12 (12.0%)	0	49 (100%)
1 tOPV + 1 bOPV + 1 IPV	0	0	0	5 (10.6%)	0	0	0

Abbreviations: mOPV2, monovalent oral polio vaccine type 2; nOPV2, novel oral polio vaccine type 2; bOPV, bivalent oral polio vaccine type; tOPV, trivalent oral polio vaccine type; IPV, inactivated polio vaccine; Sd, standard deviation.

Figure 1. Differences in the distribution of poliovirus type 1 and 3-specific stool IgA MFIs at baseline and two weeks after monovalent polio vaccine type 2 challenge. Colours and symbols indicate the polio vaccine received as a challenge. *P*-values are from Wilcoxon matched-pairs signed-rank tests comparing the distributions of $\log_{10}$ IgA MFIs between baseline and 2 weeks after the challenge. Abbreviations: PV1, Poliovirus type 1; PV3, Poliovirus type 3; mOPV2, monovalent oral polio vaccine type 2; nOPV2, novel oral polio vaccine type 2; IgA, Immunoglobulin A; MFI, median fluorescence intensity. Of note, the adults with inactivated polio vaccine (IPV) vaccination background from the nOPV2 trial (n=17) were excluded from this analysis to maintain the comparability between the groups.

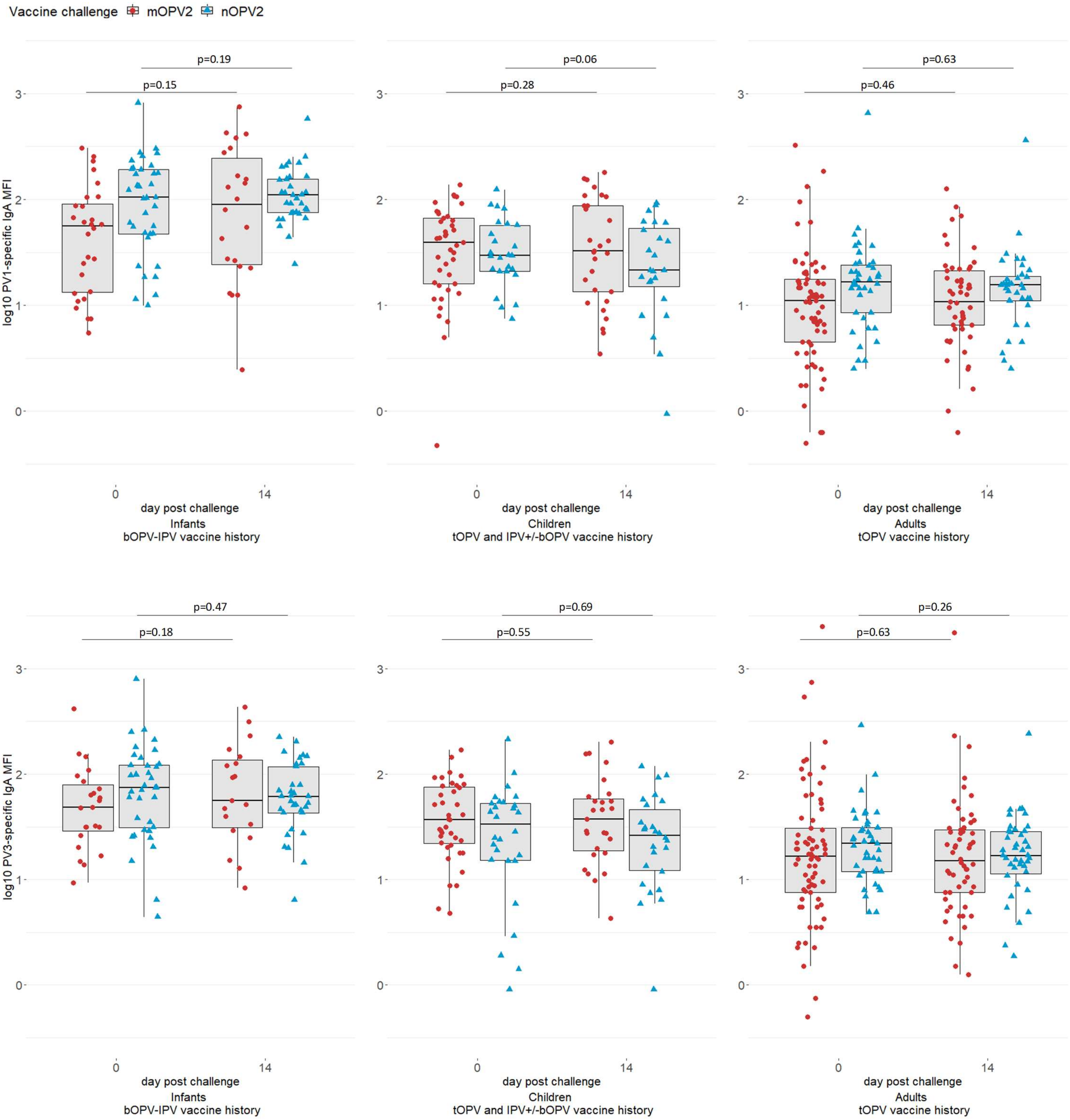

Figure 2. Correlations between serum responses, viral shedding, and markers of mucosal immunity. Colours and symbols indicate the polio vaccine received as a challenge. Abbreviations: mOPV2, monovalent oral polio vaccine type 2; nOPV2, novel oral polio vaccine type 2; PV2, Poliovirus type 2; IgA, Immunoglobulin A; CCID50, 50% cell culture infective dose; tOPV, trivalent oral polio vaccine type. Of note, the adults with inactivated polio vaccine (IPV) vaccination background from the nOPV2 trial (n=17) were excluded from this analysis to maintain the comparability between the groups.

A. Serum neutralizing activity at baseline and mucosal responses 14 days after the challenge

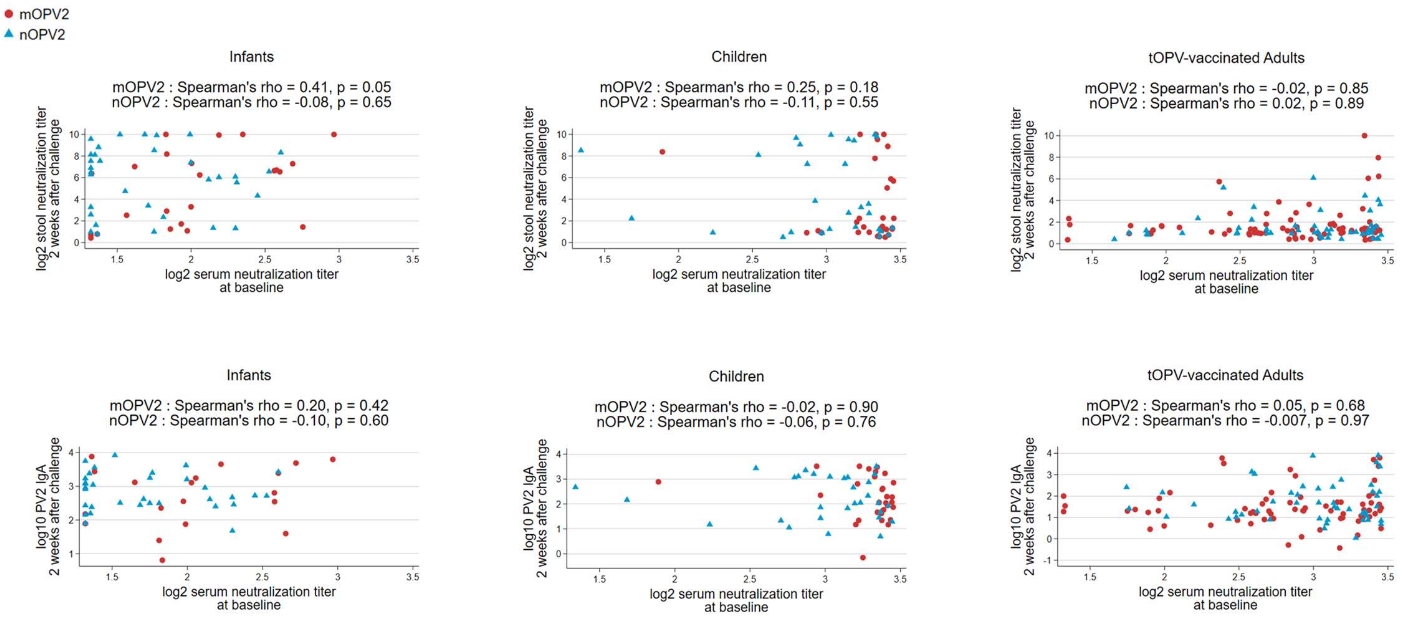

B. Shedding 7 days after the challenge and mucosal responses 14 days after the challenge

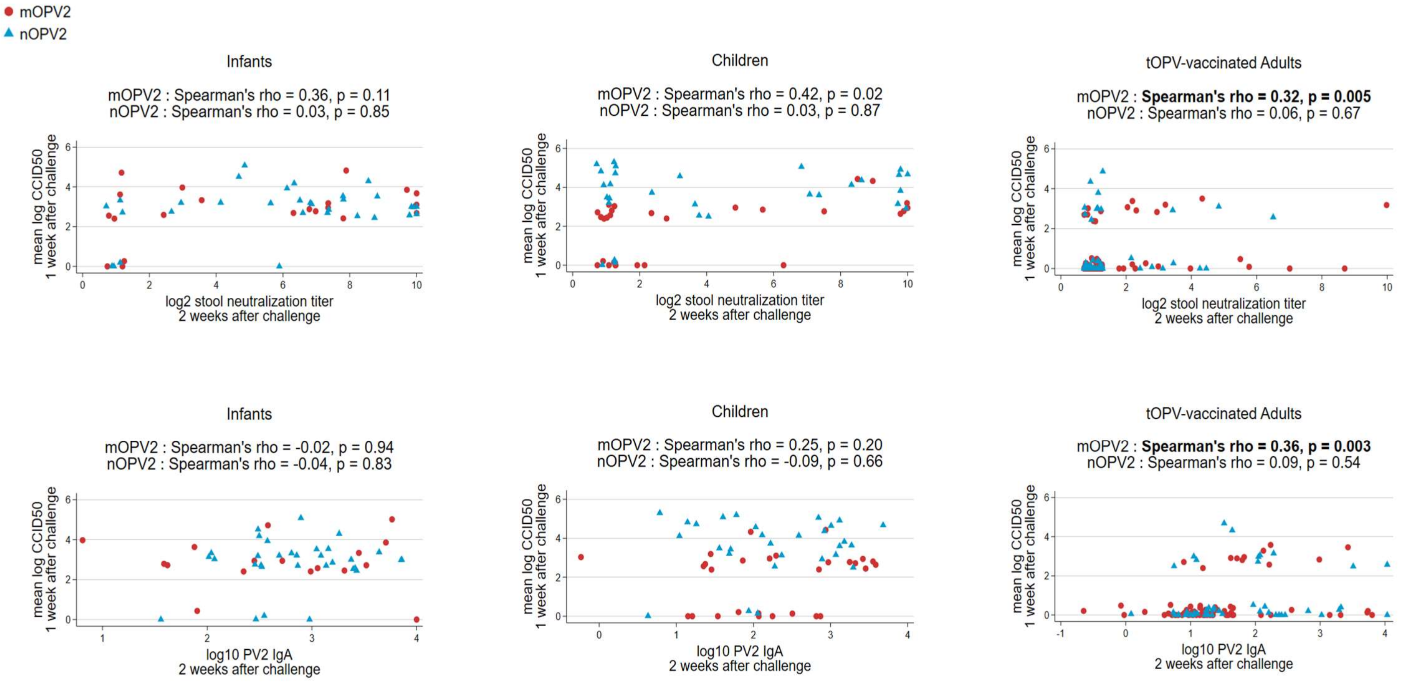

C. Stool neutralizing activity and IgA responses 14 days after the challenge.

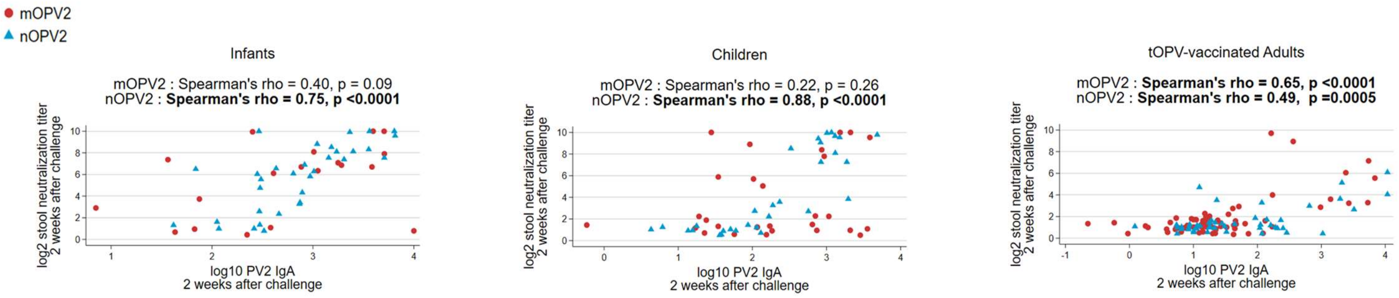

Supplement: Supplementary appendix [file mmc1.pdf]
